# The XVI International Medical Congress at Budapest in 1909

**Published:** 1907-05

**Authors:** 


### EDITORIAL NOTES AND COMMENTS.

The Business Office of the JOURNAL-RECORD is 1 1-2 to 5 1-2 South Broad Street.

The Editorial Office is 318-319-320 The Prudential.

Address all Business Commucications to Dr. George Brown, Manager.

Make remittances payable to THE JOURNAL-RECORD OF MEDICINE.

On matters pertaining to the Editorial and Original communications, address Dr, Bernard Wolff, Atlanta.

Reprints of original articles will be furnished at cost price. Requests for the same should always be made on THE MANUSCRIPT.

We will present, postpaid, on request, to each contributor of an original article, twenty 20) marked copies of THE JOURNAL-RECORD OF MEDICINE containing such article.

### THE XVI. INTERNATIONAL MEDICAL CONGRESS AT BUDAPEST IN 1909.

The Fifteenth International Medical Congress, held in Lisbon, have chosen Budapest, the capital and residence of Hungary, for the site of their next assembly, and the preliminaries are already in process.

The King has graciously taken upon himself the patronage of the ensuing congress. The state and town have each contributed 100,000 crowns to defray expenses.

The committee for the organization, execution, disbursements and reception, as also for the sections is already formed and the statutes are drawn up.

There are 1 sections, each branch of science having a separate section assigned to it.

The date of the opening is fixed for the 29th of August, 1909, and the sessions will be continued till the 4th of September.

There is every reason to presume that the congress will be well attended. Hitherto they have shown an attendance of from 3,000 to 8,000 participants. Judging from the geographical situation of

Budapest at least from 4,000 to 5,000 participants may safely be reckoned upon.

The managers, of course, attach the utmost importance to the scientific activity of the congress, and every effort is being made to win over the most prominent representatives of medical science.

The first circular, which will contain very necessary information as well as the statutes, will be ready for circulation in the course of the year 1907. Meanwhile the secretary-general of the congress (XVIth International Medical Congress, Budapest, Hungary, VIII., Esterhazy-utcz 7), will have much pleasure in giving information to inquirers.

